# Reconstructing genome-wide regulatory network of *E. coli *using transcriptome data and predicted transcription factor activities

**DOI:** 10.1186/1471-2105-12-233

**Published:** 2011-06-13

**Authors:** Yao Fu, Laura R Jarboe, Julie A Dickerson

**Affiliations:** 1 Bioinformatics and Computational Biology Program, Iowa State University, Ames, Iowa, USA; 2 Chemical and Biological Engineering Department, Iowa State University, Ames, Iowa, USA; 3 Electrical and Computer Engineering Department, Iowa State University, Ames, Iowa, USA

## Abstract

**Background:**

Gene regulatory networks play essential roles in living organisms to control growth, keep internal metabolism running and respond to external environmental changes. Understanding the connections and the activity levels of regulators is important for the research of gene regulatory networks. While relevance score based algorithms that reconstruct gene regulatory networks from transcriptome data can infer genome-wide gene regulatory networks, they are unfortunately prone to false positive results. Transcription factor activities (TFAs) quantitatively reflect the ability of the transcription factor to regulate target genes. However, classic relevance score based gene regulatory network reconstruction algorithms use models do not include the TFA layer, thus missing a key regulatory element.

**Results:**

This work integrates TFA prediction algorithms with relevance score based network reconstruction algorithms to reconstruct gene regulatory networks with improved accuracy over classic relevance score based algorithms. This method is called Gene expression and Transcription factor activity based Relevance Network (GTRNetwork). Different combinations of TFA prediction algorithms and relevance score functions have been applied to find the most efficient combination. When the integrated GTRNetwork method was applied to *E. coli *data, the reconstructed genome-wide gene regulatory network predicted 381 new regulatory links. This reconstructed gene regulatory network including the predicted new regulatory links show promising biological significances. Many of the new links are verified by known TF binding site information, and many other links can be verified from the literature and databases such as EcoCyc. The reconstructed gene regulatory network is applied to a recent transcriptome analysis of *E. coli *during isobutanol stress. In addition to the 16 significantly changed TFAs detected in the original paper, another 7 significantly changed TFAs have been detected by using our reconstructed network.

**Conclusions:**

The GTRNetwork algorithm introduces the hidden layer TFA into classic relevance score-based gene regulatory network reconstruction processes. Integrating the TFA biological information with regulatory network reconstruction algorithms significantly improves both detection of new links and reduces that rate of false positives. The application of GTRNetwork on *E. coli *gene transcriptome data gives a set of potential regulatory links with promising biological significance for isobutanol stress and other conditions.

## Background

Gene regulatory networks play an essential role in controlling gene expression and ensuring that the right genes are expressed or silenced at the right time in the right place to make the organism function appropriately. Better understanding of gene regulatory structure aids biological researchers and biochemical engineers in obtaining more complete views of the complex gene expression and regulatory mechanisms in organisms.

**Figure 1:**
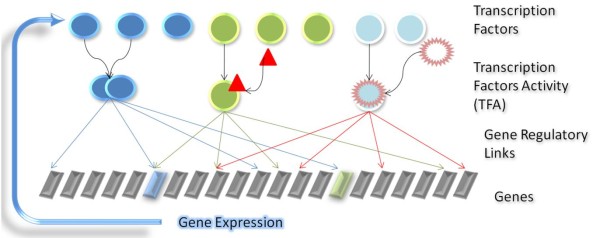
**Gene regulatory network model**. In this gene regulatory network model, a layer of Activated Transcription Factors added between the Transcription Factors layer and Gene layer. Only activated transcription factors can regulate the expression of genes through the gene regulatory links, inactivated transcription factors do not have regulatory links to the target genes. And the expression level of genes regulated by activated transcription factors changes by the effect of regulation, and the changed expression levels of genes affect the amount of the translated transcription factors.

In the gene regulation process, an active transcription factor (TF) can bind DNA and control gene expression. However, many TFs are not inherently active. Complex mechanisms, such as forming dimers, interacting with signal metabolites or binding specific micro-RNAs, are needed in order to control the activities of these TFs [1]. The activities of TFs also differ in different environments or during specific periods of cell development. This activation level is called transcription factor activity (TFA) [1] (Figure 1). Thus, TFA is an essential component of gene regulatory networks. It regulates gene expression in response to internal and external signals to ensure appropriate gene expression.

Since TFA is governed by various complex molecular interactions, it is difficult to determine directly from experiments, especially if the activation mechanism is unknown. However, it is possible to computationally predict the change of TFAs relative to a reference state using transcriptome data and a known TF-gene network architecture [1,2]. Network Component Analysis (NCA) developed by Liao et al. defines the problem of calculating TFAs as optimization of a linear least square matrix decomposition. Liao et al. solve the problem using an expectation maximization (EM) approach [3]. Fast Network Component Analysis (FastNCA) uses singular value decomposition (SVD) and a matrix projection technique to approximate the linear least square matrix decomposition problem defined in NCA[4]. Similarly, Alter and Golub use SVD and pseudo-inverse projection, and integrate ChIP and microarray data to calculate the hidden TFA layer between TFs and genes [5]. ChIP data provides additional information on proteins' DNA binding occupancy. Gao et al. developed an algorithm that combines microarray data for mRNA expression and transcription factor occupancy to define the regulatory network (MA-Networker algorithm) to predict TFAs based on ChIP and transcriptome data using multivariate regression and backward variable selection [6]. With the predicted TFAs, Gao et al. calculate the TF-gene coupling factor using Pearson Correlation [6]. Boulesteix et al. applied statistically inspired modification of the partial least square (SIMPLS) algorithm to find TFAs [7]. Many more complex models are also applied to predict TFAs. For example, Nachman et al. apply the Bayesian Network approach to provide a probabilistic model to predict TFAs [8]. The State-space model by Li et al. assumes the TFAs are affected by the TF gene expressions of previous time points [9]. Probabilistic dynamical models by Sanguinetti et al. consider the possibility of the same TF having different activities on different target genes [10]. A Gaussian process model developed by Gao et al. uses the Bayesian marginalization approach to predict TFAs [11]. Besides predicting TFAs from gene expression data and TF network structures from experiments and literature data, DNA sequence motif information is also widely used (e.g. searching for DNA binding site of TFs) in many methods to infer potential TF-gene links to obtain a more complete TF network structure and improve the prediction of TFAs [2]. However, compared to matrix decomposition and regression approaches, these complex models require more computational power. Thus, these complex models either cannot deal with large scale TFAs or they predict large scale TFAs by converting TFAs into binary.

High-throughput technologies have led to many algorithms for the reconstruction of large scale gene regulatory networks [12]. For example, many sequence analysis approaches which identify potential TF binding sites have been developed [13]. However, many of the predicted potential TF binding sites are not functional (false positive predictions) [12]. From ChIP-chip technology, potential gene regulatory effects can be derived by identifying the portions of a genome that are bound by a particular TF *in vivo *[14]. Transcriptome data (also known as gene expression data) measured by genome-wide DNA microarrays are widely used for gene regulatory network reconstructions. For instance, Stuart et al. use correlation coefficients between mRNA levels of genes as relevance scores to reconstruct correlation networks [15]. The interacting genes are predicted by detecting the correlation score above some set threshold. Other algorithms such as RELNET (RELevance NETworks) [16] and ARACNE (Algorithm for the Reverse engineering of Accurate Cellular NEtworks) [17] use mutual information as the relevance scores. The CLR (Context Likelihood Relatedness) [10] algorithm uses an adaptive background correction method on the relevance scores to improve precisions [18]. CLR significantly improved the performance of gene regulatory network reconstruction, and is widely adopted in the latest developed gene regulatory network reconstruction algorithms. In the field well known conference on Dialogue for Reverse Engineering Assessments and Methods (DREAM) [19], many winning algorithms are based on CLR. For examples, the best performer algorithm in DREAM2 Challenge 5, synergy augmented CLR (SA-CLR), introduced three way mutual information instead of the pair-wise mutual information in the original CLR [20]. Madar et al. developed a ordinary differential equation (ODE) based dynamic model extension of CLR (mixed-CLR/tl(time-lagged) CLR integrated with Inferelator 1.0) to treat steady-state data and time-series data separately and had an outstanding performance on DREAM3 and DREAM4 100-gene *in silico *network challenge [21,22]. Huynh-Thu et al. developed a regression and tree based algorithm to reconstruct gene regulatory networks and awarded the best performer in DREAM4 *in silico *Multifactorial challenge [23]. Pinna et al. developed a graph analysis based algorithm to predict directed gene regulatory network from gene knockout experiments [24].

Many gene regulatory network reconstruction algorithms focus only on time series transcriptome data to develop dynamic models [25]. These include network identification by multiple regression [26], microarray network identification [27] and multi-scale time-correlation estimation [28]. time-series network identification [29], directed information-based CLR [30]. Dynamic Bayesian network models use a Bayesian Framework to reconstruct gene regulatory networks [31,32].

Time-series based algorithms and dynamic Bayesian networks models can provide realistic models to reconstruct gene regulatory networks. However, due to a lack of closely spaced time-series data and computational power, these algorithms are difficult to apply on a genome-wide scale. Relevance score based algorithms are more efficient computationally and can integrate many different types of transcriptome data.

The standard simplified two-layer (TF-gene) model assumes a gene regulatory network model in which expressed TFs affect their target genes directly, despite the fact that TFA plays an important role in gene regulation. This simplification may lead to large false positive detection rates. Recently, the problem that TF gene expression does not necessarily correlate with target gene expression was noted in [33]. This discrepancy was addressed using a knowledge base representation of a TF expression by averaging the expressions of its target genes [33]. In our GTRNetwork model, we introduce a hidden layer of TFAs into relevance score approaches which connects TFs and their target genes. The three layer model (Figure 1) is more realistic than the two-layer model, and more biologically reasonable than the knowledge base representation model. The GTRNetwork model results in an approach to reconstruct large scale genome-wide gene regulatory networks that is both biologically more meaningful and computationally feasible.

**Figure 2:**
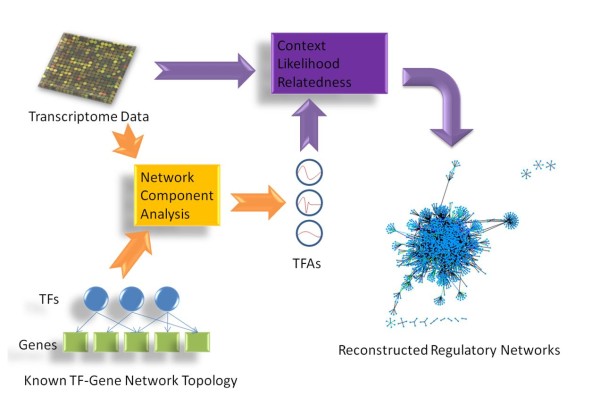
**Gene expression and Transcription factor activity based gene Regulatory Network (GTRNetwork) framework**. GTRNetwork algorithm has two steps. Step 1 (Yellow) take input of transctriptome data, predict transcription factor activities (TFAs) of TFs from known TF-Gene Network Topology. Step 2 (Purple) take the input of trancriptome data and introduce the predicted TFAs from step 1 to reconstruct gene regulatory network use score based network reconstruction methods.

The proposed **G**ene expression and **T**ranscription factor activity based **R**elevance **N**etwork (GTRNetwork) is a novel gene regulatory network reconstruction algorithm. It introduces a hidden layer of TFAs into relevance score based network reconstruction algorithms (Figure 2). The GTRNetwork combines relevance score based algorithms and TFA prediction algorithms, and generally follows two major steps. In Step 1, TFA ratios are predicted from transcriptome data and a specified TF-gene network topology. Transcript abundance ratios can be obtained from cDNA microarray or short read sequencing technology data. TF-gene network topologies can be assembled from online databases, such as RegulonDB [34]. However, TFA prediction algorithms are only based on the known TF-gene network topology and not able to predict new regulatory links. In Step 2 of GTRNetwork, gene regulatory networks are reconstructed from the gene expression ratio data and the predicted TFAs. Instead of using gene expression level as the only input to detect relationships between TFs and genes, GTRNetwork uses the relevancies between TFs and genes estimated based on the TFA and gene expression ratios. A check operon step can be used to improve the sensitivity of regulatory link detection. When gene operon information is available, it can be integrated after obtaining the reconstructed gene regulatory networks. By using gene operon information, when one gene in the operon is detected as a TF target, other genes in the same operon are automatically linked to the same TF.

## Results

### Selection of TFA prediction algorithms and network reconstruction algorithms

Different TFA prediction algorithms and network reconstruction algorithms affect the performance of the GTRNetwork method. In this research, the task is to reconstruct gene regulatory networks of *E. coli *in the whole genome scale, which includes over 4000 genes and 160 TFs. In TFA prediction algorithms, only the algorithms using matrix decomposition and regression approaches could fit the computational requirements and scale needs of GTRNetwork algorithm for a whole genome. Three major approaches to predict TFAs are: gNCA-r which uses expectation maximization (EM) [3], FastNCA which uses singular value decomposition (SVD) [4], and SIMPLS which uses partial least square (PLS) regression [7].

Similar scale and computational power requirements as the TFA prediction algorithms exist in regulatory network reconstruction algorithms using TFAs and gene expression levels. The relevance scores are calculated by either Pearson correlation coefficients or adaptive partitioning mutual information (APMI) [35]. While using relevance scores approach on microarray experiments, different genes may have different background noise in different patterns and scales. For example, relevance scores may fail to distinguish direct interaction from indirect influences when the experimental conditions are unevenly sampled, or when the microarray normalization fails to remove false background correlations [18]. Research by Faith *et al*. [18] showed that using a background correction in the relevance score based network reconstruction process reduces the false positive detection rate of regulatory links and significantly improves the performance of the network reconstruction. The Context Likelihood Relatedness (CLR) [18] algorithm provides background correction on relevance scores in GTRNetwork.

### GTRNetwork Algorithm Testing

The performance of the GTRNetwork algorithm using different combinations of TFA prediction algorithm and relevance score based network inference algorithms have been tested. Three TFA prediction algorithms (EM-based gNCA-r, SVD-based FastNCA, and regression-based SIMPLS) and two relevance score functions (Pearson correlation coefficient and adaptive partitioning mutual information) have been tested with or without using CLR background correction. The GTRNetwork algorithm using the expression level of TFs as TFAs was also tested to demonstrate its performance without including the TFA layer. Detailed information on the tested algorithms can be found in Table 1.

**Table 1:** GTRNetwork Algorithm Combinations.

GTRNetwork Algorithm Variant	TFA prediction	Relevance score	CLR Background correction
**E-A-C**	EM	APMI	Yes
**E-A-N**	EM	APMI	No
**E-C-C**	EM	Cor	Yes
**E-C-N**	EM	Cor	No
**P-A-C**	PLS	APMI	Yes
**P-A-N**	PLS	APMI	No
**P-C-C**	PLS	Cor	Yes
**P-C-N**	PLS	Cor	No
**S-A-C**	SVD	APMI	Yes
**S-A-N**	SVD	APMI	No
**S-C-C**	SVD	Cor	Yes
**S-C-N**	SVD	Cor	No
**N-A-C**	None	APMI	Yes
**N-A-N**	None	APMI	No
**N-C-C**	None	Cor	Yes
**N-C-N**	None	Cor	No

GTRNetwork algorithms using different combination of TFA prediction algorithm and relevance score based network inference algorithms.

To test the performance of the GTRNetwork algorithm using TF-gene network topologies providing different levels of information as inputs, the training datasets of input initial TF-gene network topologies are obtained by randomly knocking out 70%, 50%, 30% or 10% of links from the TF-gene regulatory links dataset of RegulonDB 7.0 [34]. The testing datasets of TF-gene networks are the links that have been removed from the training datasets respectively. Thus, the ability of the algorithm to predict the removed regulatory links is tested. The transcriptome data input for testing the GTRNetwork algorithm is an *E. coli *gene expression data set integrating 466 transcriptome experimental conditions on 4279 gene probes from the M3D database [36]. The operon information was downloaded from the RegulonDB 7.0 database [34] and used in the check operon step to find more regulatory links. GTRNetwork algorithms were applied to the input training datasets to reconstruct gene regulatory networks with different network sizes. The results are compared with the testing datasets described above and the precision and recall (sensitivity) values are calculated for each network:(1)(2)

**Figure 3:**
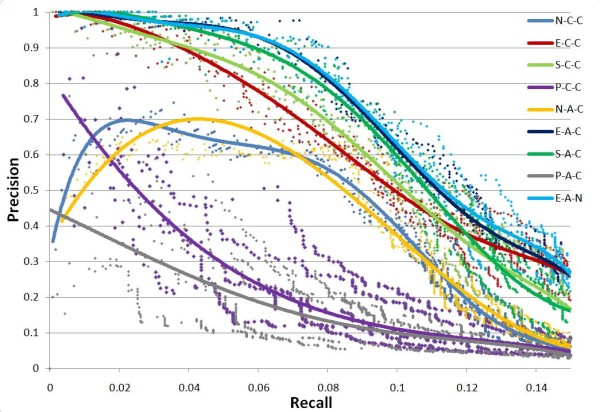
**GTRNetwork algorithm combinations on input initial network of 30% RegulonDB 7.0 data**. 70% of links are randomly deleted. Five runs were made for each recall level. The trend lines of data points are fitted by polynomial functions. Under this condition the combination E-A-C (EM-based TFA prediction, APMI relevance score with CLR background correction) and E-A-N (EM-based TFA prediction, APMI relevance score without CLR background correction) give the best performances. All the TFA based algorithms except the SIMPLS based TFA prediction show significantly better performance than the algorithms not using TFA information.

**Figure 4:**
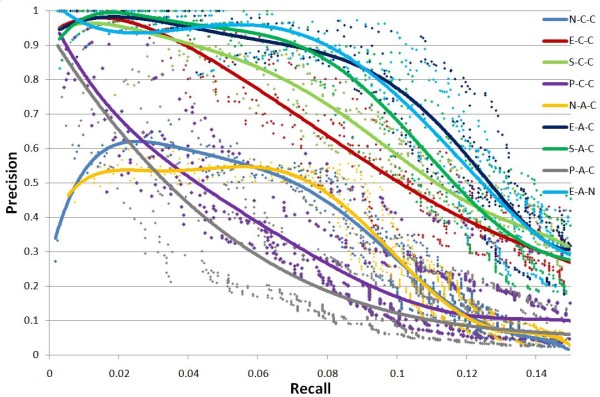
**GTRNetwork algorithm combinations on input initial network of 50% RegulonDB 7.0 data**. 50% of links are randomly deleted. Five runs were made for each recall level. The trend lines of data points are fitted by polynomial functions. Under this condition the combination E-A-C (EM-based TFA prediction, APMI relevance score with CLR background correction) and E-A-N (EM-based TFA prediction, APMI relevance score without CLR background correction) give the best performances. All the TFA based algorithms except the SIMPLS based TFA prediction show significantly better performance than the algorithms not using TFA information. At the low recall levels, the regression based TFA prediction algorithms (P-C-C and P-A-C) have better performance than the algorithms not using TFA information while.

**Figure 5:**
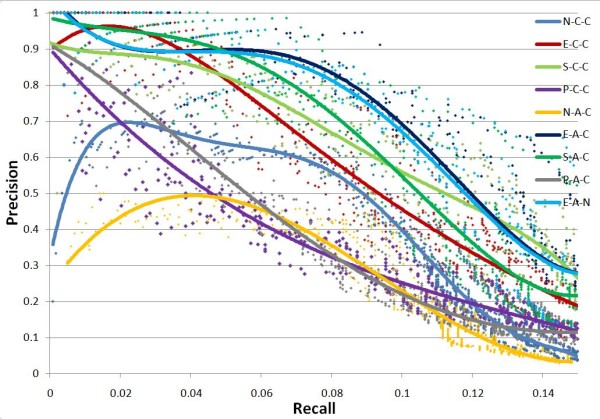
**GTRNetwork algorithm combinations on input initial network of 70% RegulonDB 7.0 data**. 30% of links randomly deleted. Five runs were made for each recall level. The trend lines of data points are fitted by polynomial functions. Under this condition the combination E-A-C (EM-based TFA prediction, APMI relevance score with CLR background correction) and E-A-N (EM-based TFA prediction, APMI relevance score without CLR background correction) give the best performances. All the TFA based algorithms except the SIMPLS based TFA prediction show significantly better performance than the algorithms not using TFA information. At the low recall levels, the regression based TFA prediction algorithms (P-C-C and P-A-C) have better performance than the algorithms not using TFA information.

**Figure 6:**
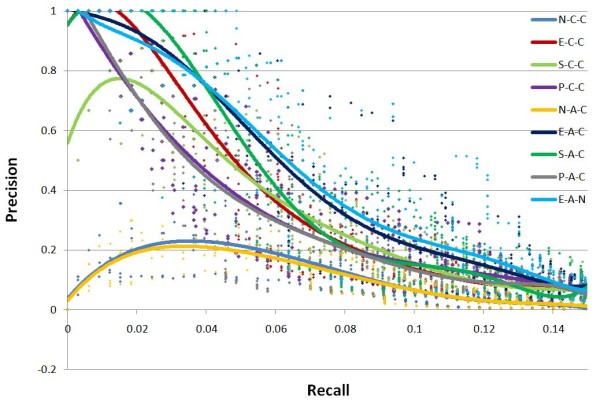
**GTRNetwork algorithm combinations on input initial network of 90% RegulonDB 7.0 data**. 10% of links are randomly deleted. Five runs were made for each recall level. The trend lines of data points are fitted by polynomial functions. Under this condition the combination E-A-C (EM-based TFA prediction, APMI relevance score with CLR background correction) and E-A-N (EM-based TFA prediction, APMI relevance score without CLR background correction) give the best performances. All the TFA based algorithms show significantly better performance than the algorithms not using TFA information.

**Figure 7:**
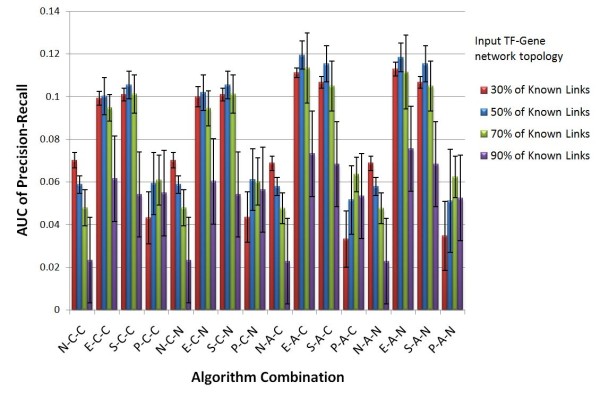
**Area under curve of precision-recall (AUCPR) of GTRNetwork algorithm combinations with different input TF-gene network topologies**. The performance of GTRNetwork is relatively consistent while using input TF-gene network topologies containing different percentages of known regulatory links, except using the 90% of known regulatory links as the input TF-gene network topology. EM-based or SVD-based TFA prediction algorithms (E/S-C-C, E/S-C-N, E/S-A-C, E/S-A-N) give significantly better performance than algorithms without using TFA information (N-X-X) or algorithms using PLS based TFA prediction (P-X-X). The algorithms using APMI relevance score function (the right half of the plot) show slightly better performance than the algorithms using Pearson correlation relevance score function (the left half). And there are no significant differences due to the use of the CLR background correction (X-X-C or X-X-N).

On each percentage level of input training dataset, the test is repeated five times to estimate the stability of GTRNetwork algorithms. In the Precision-Recall plots, all algorithm combinations show the same trend: as recall value increases, precision decreases. (Figures 3, 4, 5 and 6). At the same recall level, higher precision suggests better performance of the algorithm; while at the same precision, the larger recall value shows better performance of the algorithm. And the area under precision-recall curve (AUPRC) for each test are calculated (Figure 7). The larger AUPRC value tells us the better performance. The test results for all combinations of the GTRNetwork algorithm are shown in Additional file 1.

**Figure 8:**
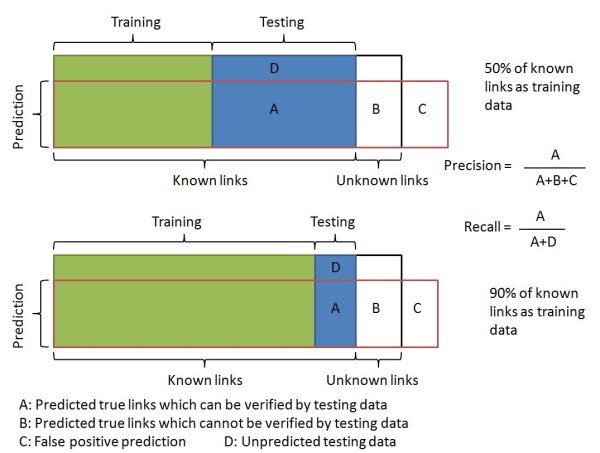
**Demonstration of TF-Gene regulatory links data**. The prediction (the area in red line) includes a part of the training data, a part of the testing data, a part of currently unknown links and some false positive predictions. When the percentage of known links as training data increases, since more training data is used, at the same recall level, the false positive decreases, and the precision (portion of area A in the area A+B+C) decreases.

There are four factors which affect the performance of GTRNetwork: the TFA prediction algorithm, the relevance score function, the background correction effect, and the network sizes of initial TF-gene network topology. Figure 7 shows that using predicted TFA information from EM or SVD-based method significantly improved the performance of the gene regulatory network reconstruction. (Two sample t-test p-value < 0.0001). The APMI relevance score function gives slightly better performance than the correlation relevance score function. (Paired two sample t-test p-value < 0.0001). However, there is no clear difference between using or not using the background correction of CLR. (Paired two sample t-test p-value = 0.8342). The performance of most algorithm combinations is relatively consistent while using different level of known knowledge of the initial TF-gene network topologies. However, when using the 90% of known TF-gene links as the initial network topology, the performances drops significantly. This performance drop is expected because as the training data (the portion of known TF-gene links) increases, the testing data is reduced. Many predicted links are already known, and only few links can be identified as new predicted links. Also many new predicted links might not be included in the testing dataset thus not being verified as a true positive prediction. However, the unverified prediction could still be true since the testing dataset is not a complete dataset; our knowledge of the complete biology of this system is still incomplete. When the portion of the known TF-gene links is increased in the training data, the total number of predicted new links decreases. At the same time, the number of unknown regulatory links in prediction would not change, or even increase because of more complete training information. Thus, the portion of unknown regulatory links in the prediction is increased. In this case, the testing is closer to a prediction. The verification based only on known knowledge cannot reflect the real performance of identifying potential new gene regulatory targets (Figure 8).

In conclusion, the algorithms using EM-based or SVD-based TFA prediction methods along with the APMI relevance score gave the best performance. In general, using or not using CLR background correction does not give significant differences in performance, but since CLR has low computational requirements (See the discussion session) and has been shown helpful in gene regulatory reconstruction algorithms [18], we suggest the use of CLR background correction in the GTRNetwork algorithm. Thus, the E-A-C (EM-based TFA prediction, APMI relevance score function and using the CLR background correction) combination is used as the default GTRNetwork algorithm in the testing and application below.

**Figure 9:**
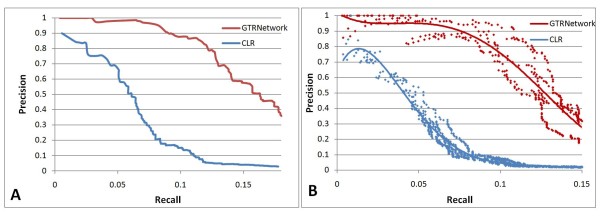
**Comparison between GTRNetwork and CLR on *E. coli *data**. **(A) **Precision-recall curve of testing results of GTRNetwork and CLR algorithms using transcriptome data from M3D database [36] and the input training TF-Gene topology of the full set of RegulonDB 7.0 [34]. **(B) **Precision-recall plot of testing results of GTRNetwork and CLR algorithms using transcriptome data from M3D database [36] and the input training TF-Gene topology of 50% links randomly knocked out RegulonDB 7.0 [34] data. Five random replications are applied on the test. The precision and recall are calculated based on the testing data of the knocked out RegulonDB 7.0 [34] on each replication respectively. The trend lines are fitted by polynomial functions.

A comparison between the original CLR [18] and GTRNetwork algorithm is also applied on the M3D *E. coli *data (Figure 9). Comparisons between CLR algorithm and many other gene regulatory network reconstruction algorithms have been done in the CLR paper [18]. And many DREAM winning algorithms, e.g. SACLR [20] and GENEI3 [23], have compared themselves with CLR on the M3D *E. coli *data and found comparable performance with CLR [20,23]. GTRNetwork outperforms CLR significantly when we use the full TF-gene regulatory information from RegulonDB 7.0 as the initial TF-gene network topology (Figure 9A). However, the result is predictable since GTRNetwork uses the additional information of TF-gene links as input, and all other algorithms only use the list of TFs as input. While using a 50% randomly knocked out TF-gene regulatory links from RegulonDB 7.0 as the training initial TF-gene network topology, and the removed regulatory links in the training dataset as the testing data, this situation would be more relative to a real biological application. In most biological cases, only limited TF-gene regulatory information is known, and the task of gene regulatory network reconstruction algorithms is to identify new regulatory links. The result still shows stronger performance of the GTRNetwork algorithm on the task of identifying new regulatory networks based on known knowledge of gene regulatory networks (Figure 9B).

### Application of GTRNetwork Algorithm

According to the test results above, the E-A-C algorithm combination best fits the current known gene regulatory network topology from RegulonDB 7.0. This algorithm combination was applied using the full set of RegulonDB 7.0 TF-gene links as the initial network topology. The gene expression data of *E. coli *integrating 466 transcriptome experiment conditions on 4279 gene probes from the M3D database was used as the transcriptome data input. Resulting gene regulatory networks with sizes ranging from 100 links to 600 links were reconstructed. Different relevance score thresholds were set to reconstruct gene regulatory networks with different sizes. Higher thresholds result in smaller regulatory networks with fewer false positives. Lower thresholds give more complete networks, but with more false positives. A check operon step using operon information from RegulonDB 7.0 was applied to improve the sensitivity of the reconstructed regulatory networks. The complete detailed predicted results are shown in Additional file 2.

In the reconstructed 100-link regulatory network, there are three new predicted regulatory links: DicA-*insD*, DicA*-intQ*, DidA*-ydfE*. These new links are biologically verifiable since *insD, intQ *and *ydfE *are in the same operon with a TF binding site of regulator DicA, according to the binding-site information obtained from RegulonDB 7.0 [34]. In the reconstructed 200-link regulatory network, besides the three new links predicted in the 100-links network, another 13 new regulatory links were predicted (Table 2). Evidence of biological validity of 8 of these 12 new links can be found in the literature or in databases such as EcoCyc [37]. For example, IscR is an iron-sulfur cluster regulator [38] and *fdx, hscA, hscB *and *iscX *are all involved in the iron-sulfur cluster assembly process.

**Table 2:** Valid search of 12 predicted new links using literature.

TF	Gene	Supporting Evidence
**DicA**	*insD*	TF binding site verified (RegulonDB) [34]
**DicA**	*intQ*	TF binding site verified (RegulonDB) [34]
**DicA**	*ydfE*	TF binding site verified (RegulonDB) [34]
**DcuR**	*pepE*	Involved in anaerobic respiration related process (EcoCyc [37])
**Fur**	*ybdB*	*ybdB *(*entH*) is proposed to be regulated by Fur (EcoCyc [37])
**Fur**	*yncE*	*yncE *is de-repressed by Fur [41]
**IscR**	*fdx*	Some evidence that the Fdx functions as an intermediate site for Fe-S cluster assembly [42]
**IscR**	*hscA*	HscA is required for the assembly of Fe-S clusters [43,44]
**IscR**	*hscB*	HscB is a co-chaperone that stimulates HscA (Hsc66) ATPase activity [44]
**IscR**	*iscX*	Possibly involved in Fe-S cluster biogenesis [43]
**SgrR**	*sroA*	TF binding site verified (RegulonDB)[34]

New regulatory links of *E. coli *predicted in a reconstructed gene regulatory network of size of 200 links (includes 16 potential new regulatory links).

The 600-link reconstructed gene regulatory network contains 381 new predicted gene regulatory links, including links predicted by checking operon information. These 381 predicted links appear biologically meaningful. For instance, the ferric uptake regulator, Fur, is predicted to have links with many ferrous iron transporters and storage related genes (*efeU, bfd, bfr, efeB, efeO, ybdB (entH), ydiE, yqjH*). Many of these new predicted targets have unknown biological function, such as inner membrane protein gene, *ybaN*, and secreted protein gene, *yncE*. The fact that these genes may be part of the Fur regulon suggests that their function may be related to iron uptake (Table 3).

**Table 3:** Predicted Fur target genes

Gene	Gene function
***efeU***	Ferrous iron permease component of the EfeUOB ferrous iron transporter.
***ybdB (entH)***	Thioesterase that is involved in the biosynthesis of enterobactin.
***bfd***	Bacterioferritin-associated ferredoxin; predicted redox component complexing with Bfr in iron storage and mobility [2Fe-2S]
***bfr***	Iron storage protein.
***efeB***	Deferrrochelatase, periplasmic; inactive acid inducible low-pH ferrous ion transporter EfeUOB; periplasmic acid peroxidase; heme cofactor.
***efeO***	Inactive acid-inducible low-pH ferrous ion transporter EfeUOB; acid-inducible periplasmic protein.
***ybaN***	Inner membrane protein, DUF454 family, function unknown.
***ydiE***	Function unknown, hemin uptake protein HemP homolog
***yncE***	Secreted protein, possible role in iron acquisition.
***yqjH***	NADPH-dependent ferric reductase containing FAD, covalently bound to a cysteine sidechain.

Genes predicted as the targets of regulator Fur in the reconstructed regulatory network of size of 600 links. Gene function information is downloaded from the EcoGene database [45].

Despite the fact that *E. coli *is so well-characterized, there are still many genes that have no known regulators. The GTRNetwork predictions help discover the regulators of those genes still have no known regulators. In the 381 predicted links, there are 171 predicted target genes which previously had no known regulators (Additional File 2).

The reconstructed gene regulatory networks with potential new gene regulatory links can be used again in the application of predicting TFAs and identify more significantly changed TFAs in response to the experiment condition changes. For example, Brynildsen *et al*. used the gene regulatory network obtained from RegulonDB and NCA to predict TFAs of *E. coli *under isobutanol stress from transcriptome data and identified 16 significantly changed TFAs in response to the isobutanol condition [39]. We reanalyzed their transcriptome data using our reconstructed gene regulatory network, including the 381 predicted new links. This additional of the new regulatory links resulted in another 7 significantly changed TFAs in response to the isobutanol condition (Table 4).

**Table 4:** Significantly changed TFAs under isobutanol condition predicted by GTRNetwork reconstructed gene regulatory network The reconstructed gene regulatory network includes 381 potential new regulatory links, the 16 significantly changed TFAs predicted by original RegulonDB data from Brynildsen's paper [39] are not included.

TF	Function	Target Genes
**ArgR**	Arginine catabolism	***argA***, ***gltF***, argE, ***argH***, rimP, ***rbfA***, truB, ***rpsO***, ***pnp***, ***nusA***, ***infB***, ***hisP***, ***gltD***, ***gltB***, ***carB***, artP, artI, ***artQ***, artM, ***artJ***, ***hisJ***, ***hisQ***, metY, astE, astB, astD, astA, astC, hisM, argB, argC, argD, argF, ***argG***, ***argI***, argR, **carA**
**AscG**	**A**rbutin-**s**alicin-**c**ellibiose transport and utilization	***ascB***, *ascF*, ***ascG***, ***htpG***, *prpR*, ***clpB***, ***dnaJ***, ***dnaK***, *tpke11*, *groL*, *groS*, ***grpE***, ***hslU***, ***hslV***, ***ybbN***, ***lipB***, ***ybeD***, ***lnt***, ***ybeX***, *ybeY*, ***ybeZ***
**CysB**	Novobiocin resistance, sulfur utilization, and sulfonate-sulfur catabolism	*tauA, tauB, tauC, ssuC, ssuD, ssuA, ssuE, **hslJ**, **cbl**, tauD, ssuB, cysP, cysU, **cysW**, **cysN**, **cysM**, **cysK**, **cysJ**, **cysI**, **cysH**, **cysD**, cysC, **cysB**, **cysA, gsiA, gsiB, gsiC, gsiD, iaaA**, yciW**, ydjN, yeeD, yeeE***
**Lrp**	Leucine-responsive regulatory protein	*lhgO, alaT, alaU, alaV, gltT, gltU, gltV, gltW, ileT, ileU, ileV, **micF**, rrfA, rrfB, rrfC, rrfD, rrfE, rrfG, rrfH, rrlA, rrlB, rrlC, rrlD, rrlE, rrlG, rrlH, rrsA, rrsB, rrsC, rrsD, rrsE, rrsG, rrsH, rrfF, thrV, csiD, ilvX, adhE, **aroA**, fimA, fimC, fimD, fimE, fimF, fimG, fimH, gabT, **gcvH**, **gltB**, **gltD**, ilvA, **ilvD**, ilvE, ilvH, ilvH, ilvI, **ilvM**, kbl, **livF**, **livG**, **livH**, livJ, **livK**, **livM**, lrp, lysU, **malT**, ompC, **ompF**, **oppA**, **oppB**, **oppC**, **oppD**, **oppF**, **osmC**, **sdaA**, serA, **serC**, **tdh**, argO, ilvL, gabD, gabP, **osmY**, **hdeA**, **hdeB**, yhiD, dadA, **dadX**, **gcvT**, **gltF**, **stpA**, gcvP, **aidB**, fimI, yeiL, yojI, **gdhA, ilvG_1, ilvG_2, thrA, thrB, thrC**, thrL*
**MarA**	Multiple antibiotic resistance	*pqiB, pqiA, **ybaO**, nfsB, **micF**, **slp**, dctR, **acrB**, acrA, marB, marR, **marA**, **inaA**, rfaY, rfaZ, yhiD, **hdeB**, **hdeA**, **rob**, **zwf**, **fumC**, fpr, nfo, **poxB**, purA, putA, **sodA**, tolC, **ygiA**, ygiB, **ygiC**, ltaE, ybjT, **talA**, **tktB**, phr, **ybgA**, yhbW*
**MetJ**	Methionine biosynthesis and transport	*metF, **metK**, metL, **metR**, yeiB, **folE**, ahpC, ahpF, metQ, metN, metI, **metA**, metB, **metC**, metE*
**NadR**	NAD biosynthesis	***nadA, pnuC, pncB, nadB***

Bolded genes have significant changes in expression according to Brynildson's paper [39]. Underlined genes are predicted new regulatory target genes of the TF from GTRNetwork.

## Discussion

In the result section, the tests of combinations of algorithms for GTRNetwork focused on finding the best algorithm combination to give the most precise prediction and maximum recall. The test results showed that the introduction of TFA improved the prediction precision and recall rate of relevance score based gene regulatory network reconstruction significantly. The best combinations of TFA predict algorithm and relevance score functions, in terms of precision and recall depend on the sizes of the known initial TF-gene network topologies.

Besides precision and recall of predictions, other properties such as the run times of algorithms are important. Among the TFA prediction algorithms, the SVD-based, FastNCA algorithm is the fastest one. FastNCA (SVD) is 280 to 440 times faster than SIMPLS (PLS) and gNCA-r (EM) (Table 5). APMI takes about 1740 seconds to generate the relevance score matrix, while using correlation as the relevance score gets the score matrix over 1000 times faster (Table 5). Applying CLR background correction finishes in seconds but can improve the precision of the reconstructed network [18]. Thus, the most time efficient algorithm combination of GTRNetwork is the SVD-Correlation-CLR background correction (S-C-C) combination. Although under some conditions, S-C-C does not perform as well as other combinations, it provides a quick estimation with relatively reliable results. This algorithm combination could be used to quickly generate a general view of the network.

**Table 5:** Algorithm run time tests

Algorithm	PLS	EM	SVD	APMI	Correlation
Run time (seconds)	2750	1750	6.2107	1740	1.4086

The run times of the three TFA prediction algorithms PLS, EM, SVD, and the two relevance score functions: APMI and Correlation.

Input: Gene expression data: M3D *E. Coli *microarray experiments. 466 experiment conditions and 4279 gene probes. TF-gene network topology: RegulonDB 6.7 gene regulatory network with 3989 regulatory links.

Machine: CPU Intel(R) Core(TM) i7 950 @3.07GHz. RAM: 6.00 GB. OS: Windows 7 Professional 64-bit.

The algorithm combinations that use regression-based SIMPLS to predict TFAs are not as precise as the other combinations. However, SIMPLS does not have as many restrictions as NCA algorithms have, such as the non-redundancy and full column and row rank of the initial network topology. Thus, SIMPLS does not discard as much information while preprocessing data to fit the input criteria. Studies show that it can predict regulatory links that gNCA-r and FastNCA could not [7]. This property of SIMPLS is especially important when there are some regulators or genes of interest, but other TFA prediction algorithms delete these interesting regulators or genes to fit the NCA criteria (detail in Methods session). There is no optimal combination of algorithms for GTRNetwork; instead, the user needs to choose the appropriate algorithm combination based on their input data and other requirements.

The TFA prediction model does not need any biological knowledge on the detailed mechanisms of the activation of TFs. The model assumes that all of the complex effects that contribute to the change of TFA are included in the predicted TFAs and the control strengths. Thus, the GTRNetwork algorithm is not limited to prokaryotes, but can also be applied to eukaryotes. We plan to apply this method to eukaryotes such as yeast and plants in the near future.

While most relevance score based gene regulatory network reconstruction algorithms are not able to identify the self regulation of TFs, because the gene expression data is directly used as the only input to represent both the regulators and the targets, there are always high relevance scores to connect the TF and its gene. In GTRNetwork, since the representation of the regulators (TFAs) and the representation of the targets (expression of genes, including TF genes) are well separated, the relevance score between the TF and its gene is meaningful, and the self regulation of TFs can also be identified. The prediction of self regulation of TFs improves interpretation of the cyclic structures of gene regulatory networks. Further analysis of the effect of feedforward and feedback loops is not carried out in this work but will be applied on the reconstructed networks in our future work.

TFA prediction methods are all based on a linear static model of experimental conditions, and treat dynamic time series data as static data of each time point. Thus, although time series transcriptome data can be used as an input of GTRNetwork, the algorithm does not take advantage of dependencies in time series data.

## Conclusion

The algorithm GTRNetwork introduces the hidden layer TFA into classic gene regulatory network reconstruction networks. A comparison of the performances of several algorithmic variants of this algorithm showed that the E-A-C variant of the GTRNetwork use EM-based TFA prediction method, adaptive partitioning mutual information as the relevance score function and CLR background correction method. This is the variant best fits the current known TF-gene regulatory networks from RegulonDB. The application on the E-A-C variant on *E. coli *data shows a promising amount of biological significance. It would be interesting and meaningful to verify more predicted result biologically and try other alternative TFA prediction such as the SIMPLS based methods and network reconstruction algorithms computationally. The application on other organisms such as yeast is also highly recommended to be applied in the future research.

## Methods

TFA prediction

TFA prediction is based on the following biological approximation [1]:(3)

*Er_i _*is the gene expression ratio between two experiment conditions of the *i*-th gene, *TFA*r*_j_*, *j *= 1,...,L, is a set of TFA ratios of TF *j*, which regulate gene *i*, between the same two conditions, and *CS_ij _*represents the control strength of transcription factor *j *on gene *i*. After taking the logarithm of Eq. (3) [1]:(4)

where *N *× *M *matrix [*Er*] is the relative gene expression level matrix and *L *× *M *matrix [*TFAr*] is the relative transcription factor activities, the elements *Er_ij_*(*t*) = *E_ij_*(*t*)/*E_ij_*(0) and *TFAr_kj_*(t)/*TFAr_kj_*(0), *N *× *L *matrix [*CS*] is the control strength matrix of transcription factors and genes. The gene expression model in Eq. (4) can be decomposed into matrix [*CS*] and matrix *log*([*TFAr*]) using different algorithms.

The relative gene expression level matrix [*Er*] can be obtained from transcriptome experiments such as DNA microarrays or RNAseq, and the control strength information must be initialized from the literature e.g. RegulonDB [34], Chip-on-chip experiments, and motif information (mNCA [40]). The initial matrix, *CS *is converted from the known database of gene regulatory links between TFs and genes, e.g., RegulonDB [34]. Each row represents a gene and each column represents a TF. When there is a known regulatory link between gene *i *and TF j, *CS_ij_*=1, otherwise CSij = 0.

With the input of [*Er*] and [*CS*], transcription factor activities *log*([*TFAr*]) can be estimated. There are three major approaches to estimate *log *([*TFAr*]) expectation maximization (EM) approach (e.g. gNCA-r) [3], singular value decomposition (SVD) approach (e.g. FastNCA) [4] and regression approach (e.g., SIMPLS) [7].

Note: When using gNCA-r or FastNCA to estimate *log *([*TFAr*]) matrix, log ([*Er*), [*CS*] and *log *([*TFAr*]) need to fit three criteria given below to ensure the uniqueness of the decomposition [1,3,4].

(i) The connectivity matrix [*CS*] must have full-column rank.

(ii) When a node in the regulatory layer is removed along with all of the output nodes *Er_i _*connected to it, the resulting network must be characterized by a connectivity matrix that still has full-column rank. This condition implies that each column of [*CS*] must have at least *L*-1 zeros.

(iii) The matrix, log [*TFAr*], must have full row rank. In other words, each regulatory signal cannot be expressed as a linear combination of the other regulatory signals.

### Relevance Scores

Instead of calculating relevance scores between the expression levels of two genes GTRNetwork calculates the relevance score between each TFA and each gene. Pearson correlation coefficient and mutual information are chosen as the relevance score functions:

Pearson Correlation Coefficient:(5)

where *X_ik _*is the *k*-th observation of variable *i*. and *S_ij _*is the Pearson Correlation Coefficient score between variable *i *and *j*.

### Mutual Information

Where *p*(*i,j*) is the joint probability of *i *and *j*, *p*_1_(*i*) and *p*_2_(*i*) are the marginal probabilities of *i *and *j *respectively, *S_ij _*is the Mutual Information score between variable *i *and *j*.

The Pearson Correlation (Eq. 4) performs extremely well in detecting linear relationships between two variables (genes in a set of microarray experiments), and Mutual Information (MI) (Eq. 5) has a relatively balanced performance in detecting both linear and non-linear relationships. However, most MI applications only work for discrete variables, and in this problem, both the gene expression ratio and TFA ratio are continuous variables. Adaptive partitioning [35] adjustments are applied to calculate mutual information between TFA ratios and gene expression ratios.

### Background correction

In the relevance score based network reconstruction approaches; there are tradeoffs between the link detection sensitivities and false positive detection rates [10]. One reason for the false positive detection is the simplification of the two layer gene regulatory network model. Adding the TFA layer to the classic two layer regulatory network model may solve this problem. Another reason for the false positive detections is due to the noise of gene expression data and different relatedness behaviors of TFs and genes. For example, the expression of some genes may be more stable than other genes and not tend to change much in response of different conditions, the relevance score of these genes are tend to lower, and regulatory relationships between these genes and TFs are hard to be detected, the same to TFAs. Thus, a background correction method such as context likelihood relatedness (CLR) [18] is needed.

In the CLR algorithm, along with the relevance score, the statistical likelihood of each relevance score is calculated within each variable by:(7)

where *Z_ij _*is the z-score of relevance score between variable *i *and *j *within all relevance scores with *i*, *S_ij _*is the relevance score between variable *i *and *j*,  is the average of all relevance scores with *i*. And a joint likelihood between two variables is calculated from the z-scores from Eq. (6). The methods to calculate the pseudo-z-score *Z_ij _*vary and the CLR algorithm use the following method as default [18]:(8)

By putting different thresholds on the matrix [*Z*] with elements *Z_ij _*gene regulatory networks with different sensitivities can be reconstructed by searching for gene regulatory links containing TF genes with the Z score larger than the threshold. The information of TF genes (which genes encode TFs) can be found from database such as RegulonDB [34] and EcoCyc [37].

### Integration of operon information

In the reconstructed gene regulatory network, when gene *A *is predicted to be regulated by some TFs, the other genes in the same operon as gene *A *are not always predicted to be regulated by the same TFs regulating gene *A*. However, in real gene regulatory networks, all the genes in the same operon tend to have similar behavior. The GTRNetwork algorithm uses an optional check operon step. When the operon information is available, the algorithm searches for genes in the same operon as the target gene and links these genes to the regulators of the target gene. This integration of operon information improves the detection sensitivity of regulatory links.

### GTRNetwork algorithm

The GTRNetwork algorithm is implemented using Matlab and the source code is available at:

http://vrac.iastate.edu/~afu/GTRNetwork/GTRNetwork_1.2.1.zip.

Inputs: a) Log 2 ratio transcriptome data in matrix [*Err*]

b) Initial TF-gene network topology in adjacency matrix [*C*]

c) Desired size of reconstructed regulatory network *S*

d) List of operons and the genes contained in them (Optional)

Outputs: A list of predicted regulatory links

The GTRNetwork algorithm uses the TFA prediction algorithm to predict TFAs from input a) and b). It then uses relevance score functions such as correlation coefficient function or APMI to calculate the relevance score between TFAs of TFs and the expression levels of all genes. A CLR background correction is applied on the relevance score matrix. And then according to the desired size of reconstructed regulatory network (input c), a threshold based on the background corrected relevance score is calculated and the gene regulatory network is reconstructed filtered by the threshold. Finally, an optional check operon step is applied to add missing predicted regulatory links in the same operon of the predicted target genes.

1. Match the genes between the matrix [*Er*] and matrix [*C*]. 

Remove unmatched genes in [*Er*] and store the reduce matrix as [*Er0*]. 

Remove unmatched TFs and genes in [*C*] and store the reduced matrix in [*C0*].

2. If the TFA prediction algorithm is gNCA-r or FastNCA, check the three criteria described in TFA prediction section and reduce the matrix [*Er0*] and [*C0*] to fit the criteria.

3. Apply TFA prediction algorithm to predict the log_2 _ratio TFA matrix [*TFA*] from matrix [*Er0*] and [*C0*].

4. Calculate the relevance score matrix [*M*] between TFAs and all expression levels of all genes from matrix [*TFA*] and [*Er*].

5. Calculate the joint statistical likelihood matrix [*Z*] of relevance score matrix [*M*] using CLR algorithm.

6. Set a threshold T for matrix [*Z*] so that there are *S *elements in [*Z*] greater than *T*. For all the TF-gene pairs having a *Z *score greater than *T*, construct a regulatory link.

7. If the operon list is available, check and add all genes in the same operon of TF target genes to the regulatory target set of the TF.

## Authors' contributions

YF developed and implemented the GTRNetwork algorithm and drafted this manuscript. LRJ and JD developed the initial concept and suggested ways to improve the algorithm and testing methods. All authors read and approved the final version of the manuscript.

## Supplementary Material

Additional file 1 **Test results of GTRNetwork Algorithm combinations, GTRNetwork algorithm using TF-gene network topologies providing different level of information as input, the input initial TF-gene network topologies are obtained by randomly deleting 70%, 50%, 30% or 10% links of the TF-gene links data from RegulonDB 7**.0 [34]. Click here for file

Additional file 2 **Potential new regulatory links of *E. coli *predicted using GTRNetwork**. Gene expression data is obtained from M3D database [36] and contains 466 transcriptome experiment conditions on 4279 gene probes. TF-gene regulatory network from RegulonDB 7.0 [34] is used as the initial known TF-gene regulatory topology input. 381 potential new gene regulatory links are predicted. The reconstructed network size can be used as a reference of confidence of the predicted links. Smaller reconstructed network sizes indicate more confidential predictions. Gene functions information is downloaded from EcoGene database [45] Click here for file
